# The natural history of sporadic non‐ampullary duodenal epithelial tumors: Can we wait and see?

**DOI:** 10.1002/deo2.9

**Published:** 2021-04-21

**Authors:** Yoshiki Sakaguchi, Yosuke Tsuji, Tetsuo Ushiku, Dai Kubota, Junichi Sato, Miho Obata, Rina Cho, Sayaka Nagao, Nobuyuki Sakuma, Naoki Tamura, Yuko Miura, Kazushi Fukagawa, Daisuke Ohki, Hiroya Mizutani, Chihiro Takeuchi, Yu Takahashi, Chihiro Minatsuki, Keiko Niimi, Nobutake Yamamichi, Kazuhiko Koike

**Affiliations:** ^1^ Department of Gastroenterology Graduate School of Medicine The University of Tokyo Tokyo Japan; ^2^ Department of Pathology Graduate School of Medicine The University of Tokyo Tokyo Japan

**Keywords:** natural history, sporadic non‐ampullary duodenal epithelial tumor, tumor progression

## Abstract

**Objectives:**

The natural history of sporadic non‐ampullary duodenal epithelial tumors (SNADETs) is poorly documented. The aim of this study was to evaluate the history of SNADETs in patients where immediate resection could not be performed.

**Methods:**

This is a single‐center retrospective study of 86 consecutive cases of SNADETs who did not undergo immediate resection and were followed‐up with upper gastrointestinal endoscopy for more than 6 months.

**Results:**

During a follow‐up period of 36.8 (6.0–613.0) months, macroscopic progression was admitted in eight (9.3%). Of these, the final histology in four was adenocarcinoma, and three cases demonstrated submucosal invasion. Rates of macroscopic progression at 150 months after detection were 11.1%, 16.7%, and 30.0% for SNADETs <5 mm, <10 mm, and ≥10 mm, respectively.

**Conclusion:**

The overall risk of SNADETs progressing to invasive cancer is low. However, changes in macroscopic size or shape of SNADETs signify a high risk of progression to invasive cancer.

## INTRODUCTION

Cancer of the small bowel is extremely rare, accounting for only 3% of all gastrointestinal (GI) malignancies.^1^, ^2^ Of these, sporadic non‐ampullary duodenal adenocarcinomas are the most common.^2^, ^3^ Most of these cancers are presumed to develop from duodenal adenomas, through an adenoma‐carcinoma sequence involving the β‐catenin pathway, similar to colorectal carcinoma.^4^, ^5^, ^6^ However, while endoscopic resection of colorectal adenomas is strongly suggested in order to prevent colorectal cancer due to a high risk of carcinogenesis,^7^ the natural history of sporadic non‐ampullary duodenal epithelial tumors (SNADETs) is poorly documented. The only pertinent previous report to date suggests that low‐grade sporadic non‐ampullary duodenal adenomas have a low risk of progression to adenocarcinoma.^8^ Thus, due to insufficient evidence concerning the natural history of SNADETs, there are currently no widely accepted guidelines concerning the surveillance, diagnosis, treatment, and prognosis of these lesions.

However, with recent advances in endoscopic therapeutic treatment, there are an increasing number of reports on the feasibility of endoscopic resection for SNADETs.^9^, ^10^ Contrary to previous documentation on the natural history of these lesions, a high incidence of cancer has been confirmed in resected specimens.^9^, ^10^, ^11^ Recently, it has become clear that histological diagnoses of duodenal tumors based on endoscopic biopsies are accurate only to a limited degree, and this may be one of the major reasons for the discrepancy in cancer risk.^12^, ^13^ However, while endoscopic resection of SNADETs is feasible, a relatively high risk of adverse events has been reported, especially in previous decades.^14^, ^15^ Due to the lack of guidelines and a high risk of adverse events, treatment of non‐cancerous SNADETs is not globally standardized; in clinical settings, methods of treatment, or whether treatment is performed at all, has often been decided at the discretion of the endoscopist. These decisions are often influenced by patients’ comorbidities and social circumstances, and in some cases a wait‐and‐see strategy for these lesions cannot be avoided. The aim of this study was to evaluate the natural history of SNADETs in patients where immediate resection could not be performed.

## METHODS

### Study design

This is a single‐center retrospective case study of all consecutive cases of SNADETs that underwent upper GI endoscopy at the University of Tokyo between January 1, 2002 and December 31, 2019.

The study complied with the Declaration of Helsinki and was begun after approval by the Research Ethics Committee of the Graduate School of Medicine and Faculty of Medicine, The University of Tokyo.

### Patients and disease definition

**FIGURE 1:**
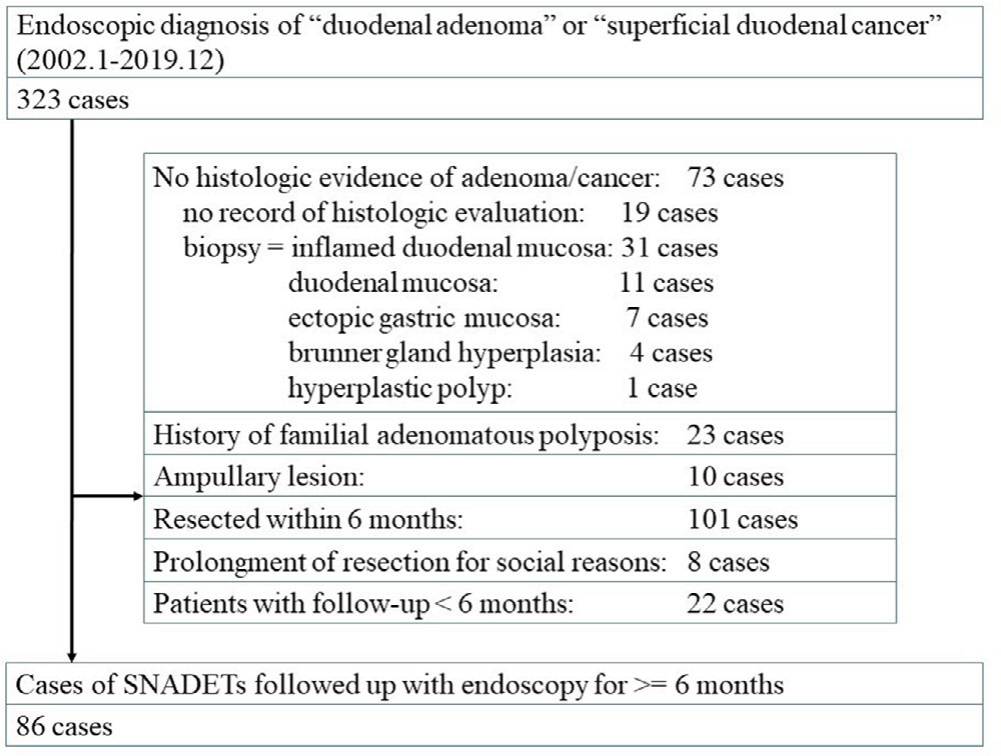
Flowchart of study selection

From the electronic medical records at the University of Tokyo Hospital, all cases who underwent upper GI endoscopy between January 1, 2002 and December 31, 2019 and were endoscopically diagnosed as either “duodenal adenoma” or “superficial duodenal cancer” were identified. A total of 323 consecutive patients were extracted. Exclusion criteria were 1) patients without histologic diagnosis of adenoma/cancer, 2) patients with a history of familial adenomatous polyposis, 3) ampullary lesions, 4) patients who underwent resection of the SNADET within 6 months, 5) patients who prolonged resection of the SNADET to more than 6 months after detection only due to social reasons, and 6) patients with a follow‐up period of less than 6 months. After exclusion, a total of 86 lesions in 86 patients who did not undergo immediate resection (endoscopic or surgical) for SNADETs within 6 months of detection and were followed‐up with upper GI endoscopy for more than 6 months were extracted and included in analysis (Figure 1).

### Evaluation of histological features

All biopsy specimens were graded according to the revised Vienna classification of gastrointestinal epithelial neoplasia.^16^ Resected specimens were histologically assessed based on criteria identical to the Japanese guidelines for cancer of the colon and rectum.^17^ In cases of referral, biopsy was performed at the University of Tokyo Hospital during endoscopic evaluation. When a histologic diagnosis of adenoma/cancer could not be reconfirmed, biopsy specimens from the referring hospital were attained when possible and assessed at the University of Tokyo Hospital. Only patients with a biopsy assessment of category 3 or above and/or a final histologic diagnosis of adenoma/carcinoma were included in analysis.

### Treatment strategy

All endoscopic examinations were performed by endoscopists with over 3 years of experience in upper GI endoscopy. All cases with an endoscopic diagnosis of either “duodenal adenoma” or “superficial duodenal cancer,” and an initial biopsy result of category 3 or above were considered indications for resection. For these cases, endoscopic images and methods of resection were discussed by a board comprised of expert endoscopists, including multiple experts in endoscopic submucosal dissection. After discussion by the endoscopic resection board, cases with endoscopic findings suggestive of submucosal invasion were referred for surgical resection, and endoscopic resection was recommended to all other cases. However, other advanced malignancies requiring immediate treatment, severe systemic illnesses, dementia, and low performance status were considered contraindications for treatment. In cases with contraindication for treatment and cases where patients refused treatment, endoscopic follow‐up was advised.

### Endoscopic examinations and biopsy during follow‐up

For patients where immediate resection was not performed, upper GI endoscopic examination follow‐up was performed at 6‐ to 12‐month intervals, and continued for as long as the patients’ medical and social circumstances allowed. Endoscopic size was assessed by comparison with endoscopic forceps size. When a change in the macroscopic shape, that is increase of thickness or depression, or an increase in size of the SNADET was endoscopically detected, endoscopic images and treatment methods were again discussed by the endoscopic resection board. When findings by the endoscopist were reconfirmed by members of the board with interobserver agreement, this was considered macroscopic progression, and immediate endoscopic resection was recommended. Endoscopic biopsy of the lesion during follow‐up was performed as required but was not a prerequisite due to the risk of severe fibrosis after repeated biopsies.^18^

### Clinical characteristics and definitions

Background factors, referral documents, endoscopic findings, and histopathological results were extracted from the medical records at the University of Tokyo Hospital. The date of detection was defined as the initial date that an SNADET was endoscopically detected either at the University of Tokyo Hospital or the referring institute. Tumor progression was based on preoperative findings and was defined as either histologic progression or macroscopic progression. Histologic progression was defined as the condition where the histologic assessment of endoscopic biopsy was upgraded (i.e., category 3 to 4 or above). Macroscopic progression was defined as an increase in the macroscopic shape or change in size of the SNADET with endoscopic examination, with interobserver agreement by multiple experts in endoscopic resection. Curative treatment by endoscopic resection was defined as R0 resection for adenomas and R0 resection with no lymphovascular invasion for intramucosal cancer. Submucosal or lymphovascular invasion were considered indications for surgical treatment. The follow‐up period was defined as the period from the date of detection to either the date of curative treatment or the last follow‐up with upper GI endoscopy.

### Statistical analysis

All continuous variables were compared using either the Student's *t* test or Mann–Whitney *U* test, and categorical variables were compared using either the χ2 test or Fisher's exact test as appropriate. Statistical significance was set at *p* value of <0.05. All statistical analyses were performed using JMP Version 15.0 (SAS Institute Inc., Cary, NC, USA).

## RESULTS

The natural history of a total of 86 lesions in 86 consecutive patients was analyzed (Table 1). Forty‐nine (57.0%) were male, and the mean age at time of detection was 65.4 ± 12.6 years. Localization of the SNADETs was 16 (18.6%), 5 (5.8%), 65 (75.6%), and 0 (0 %) in the first, superior duodenal angle, second, and third portions of the duodenum, respectively; and the mean endoscopic size of SNADETs at time of detection was 10.6 ± 8.3 mm.

**TABLE 1:** Baseline characteristics of sporadic non‐ampullary duodenal epithelial tumors

	*n* = 86
**Patient factors**	
Age	65.4 ± 12.6
Gender (M/F)	49/37
**Lesion factors**	
Macroscopic type	
0‐I	15 (17.4%)
0‐IIa	63 (73.3%)
0‐IIc	8 (9.3%)
Location	
Bulbus	16 (18.6%)
SDA	5 (5.8%)
2nd portion	65 (75.6%)
Lesion size (mm)	10.6 ± 8.3
Biopsy result	
No preoperative biopsy	1 (1.2%)
Category 1	1 (1.2%)
Category 2	0 (0%)
Category 3	80 (93.0%)
Category 4	3 (3.5%)
**Primary reason for not performing immediate resection**	
Other advanced malignancy	7 (8.1%)
Severe systemic illnesses	15 (17.4%)
Dementia, low performance status due to other reasons	15 (17.4%)
Refusal of treatment by patient for other reasons	34 (39.5%)
Unclear from clinical records	15 (17.4%)

All cases diagnosed as category 5 by endoscopic biopsy were resected, and none were included in the analysis group. Eighty‐one (94.2 %) SNADETs were classified as category 3 and 3 (3.5 %) as category 4 by preoperative endoscopic biopsy. Two cases were not classified as category 3 or above prior to treatment and were endoscopically followed‐up until macroscopic progression.

### Natural History and Tumor progression of SNADETs

**FIGURE 2:**
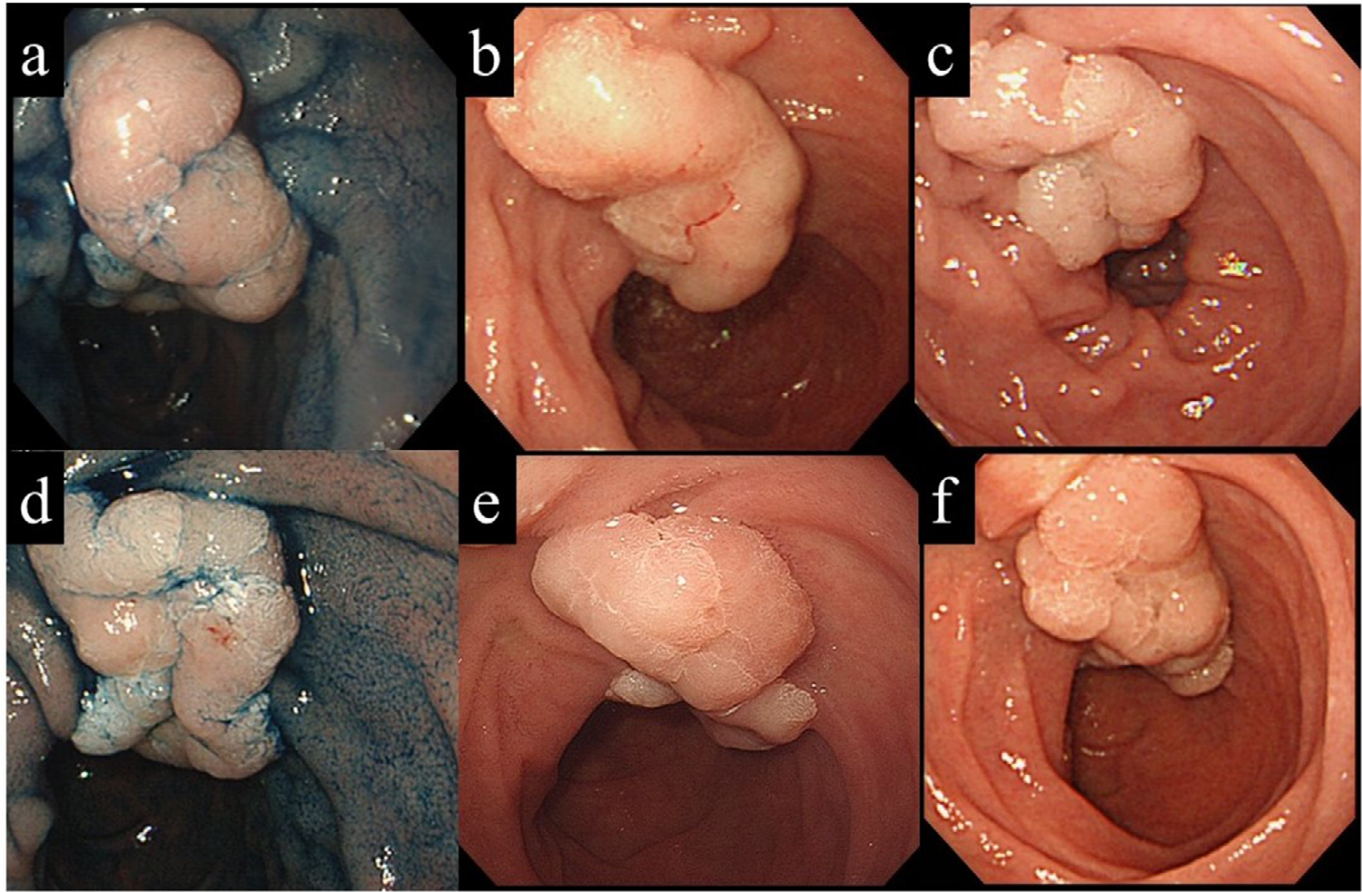
A representative SNADET case with no tumor progression during follow‐up. (a) A patient with a SNADET 4 cm in size was referred to our institute. For reasons unclear from the clinical records, the patient underwent annual endoscopic follow‐up. (b) No apparent macroscopic change was detected 1 year after referral. (c) Two years after referral. (d) Six years after referral. (e) Eight years after referral. (f) Ten years after referral. The patient was lost to follow‐up after 10 years

During a median follow‐up period of 36.8 (6.0–613.0) months, SNADETs became endoscopically and histologically undetectable in 14 (16.3%) cases, no change was admitted (Figure 2) in 62 (72.1%), and tumor progression was admitted in 10 (11.6%). All cases which became endoscopically and histologically undetectable had undergone repeated biopsies.

**FIGURE 3:**
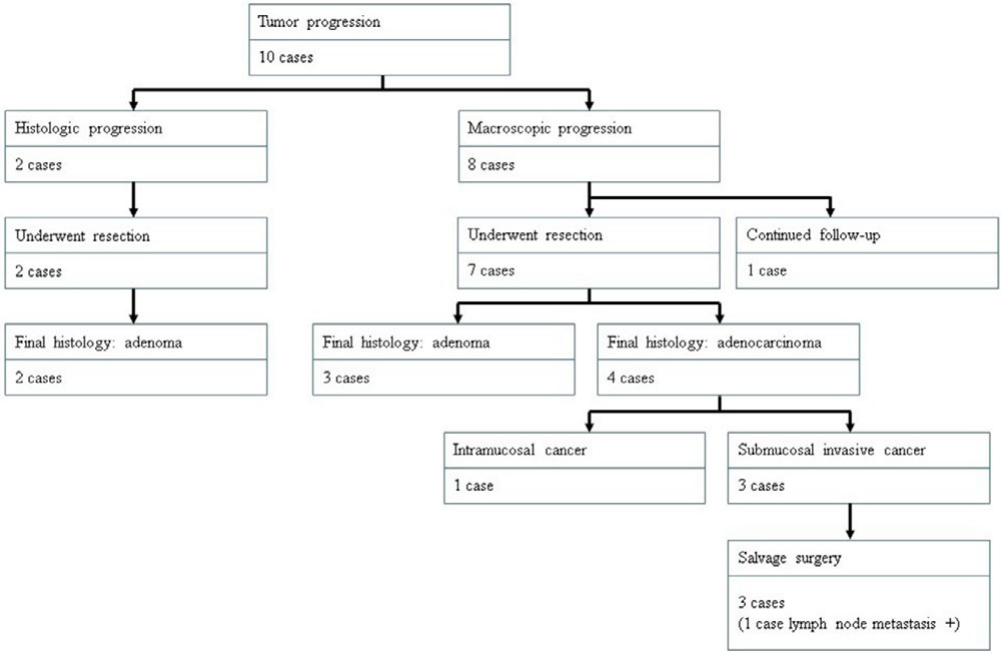
Flowchart of cases with tumor progression

**FIGURE 4:**
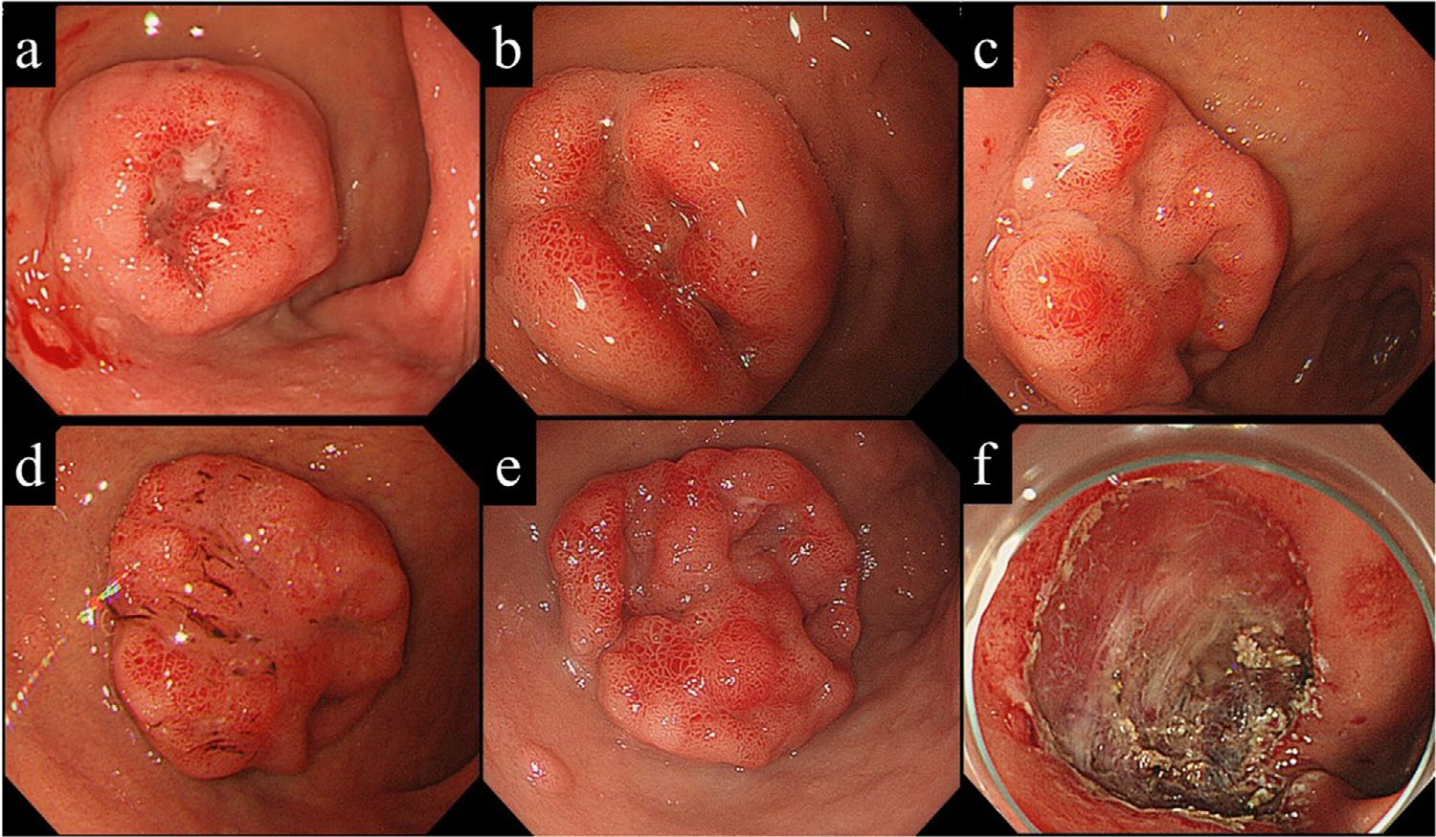
A case of tumor progression with submucosal invasion. (a) A patient with a SNADET with central depression was referred to our institute. As initial biopsy resulted in category 1, the patient requested follow‐up over resection. (b) Endoscopic follow‐up was continued, with no apparent macroscopic or histologic progression at 18 months after referral. (c) Macroscopic progression was suspected at 30 months, but with no histologic progression, the patient refused treatment and requested endoscopic follow‐up. (d) Clear macroscopic progression at 36 months with no histologic progression and after further discussion with the patient, resection was scheduled 4 months later. (e) The lesion on the day of resection displayed further changes in macroscopic shape. (f) EMR was performed at 40 months, for a final histologic diagnosis of adenocarcinoma with submucosal invasion

The median period to tumor progression was 53.4 (6.2–613.0) months, with two cases (2.3%) of histologic progression and eight cases (9.3%) of macroscopic progression (Table 2, Figures 3 and 4). The two cases with histologic progression of endoscopic biopsy from category 3 to 4 underwent resection. However, the final histology in both of these cases resulted in low grade adenoma. Among 10 cases with tumor progression, seven cases with macroscopic progression underwent endoscopic resection for a final histologic diagnosis of adenocarcinoma in four cases. Of these, three (42.9%) demonstrated submucosal invasion, and one (14.3%) had local lymph node metastasis upon salvage surgery. Following salvage surgery, no recurrences or metastases have been detected. One case demonstrated macroscopic progression at 59.7 months, but did not undergo resection, and has confirmed asymptomatic survival at 121.2 months after detection. The remaining two cases with histologic progression of endoscopic biopsy from category 3 to 4 underwent resection. However, the final histology in both of these cases resulted in low grade adenoma. Thus, the following analysis was focused on macroscopic progression.

**TABLE 2:** Characteristics of sporadic non‐ampullary duodenal epithelial tumors with tumor progression

Case	Gender	Location	Age*	Macroscopic type*	Endoscopic size*	Biopsy histology*
1	F	2nd	73	0‐IIa	10	Category 3
2	M	Bulbus	72	0‐I	10	Category 3
3	F	2nd	22	0‐I	20	Category 3
4	M	Bulbus	69	0‐I	15	No biopsy
5	F	2nd	54	0‐IIa	40	Category 3
6	F	2nd	67	0‐IIa	15	Category 3
7	M	Bulbus	54	0‐IIc	15	Category 1
8	M	2nd	67	0‐IIa	15	Category 3
9	M	SDA	72	0‐IIa	4	Category 3
10	M	2nd	65	0‐IIa	6	Category 3

Case	Type of progression	Primary macroscopic change	Months to progression	Endoscopic size at progression	Final histology	Submucosal/lymphovascular invasion
1	Macroscopic	Thickness increase	59.7	15	NA (not resected)	NA
2	Macroscopic	Thickness increase	23.7	10	adenocarcinoma	yes
3	Macroscopic	New depression	613	20	adenocarcinoma	yes
4	Macroscopic	Thickness increase	69.4	15	adenoma	no
5	Histologic	No change	136.7	40	adenoma	no
6	Histologic	No change	7.1	15	adenoma	no
7	Macroscopic	New depression	40.2	15	adenocarcinoma	yes
8	Macroscopic	Thickness increase	6.2	20	adenoma	no
9	Macroscopic	New depression	47.2	6	adenoma	no
10	Macroscopic	New depression	70.1	6	adenocarcinoma	no

* At time of detection

### Risk factors associated with macroscopic progression

**FIGURE 5:**
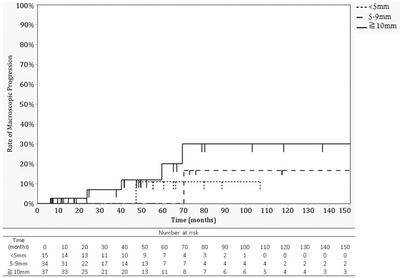
Cumulative incidence of macroscopic progression by size at detection. Kaplan‐meier curve on the cumulative incidence of macroscopic progression of sporadic non‐ampullary duodenal epithelial tumors. There was no statistically significant difference between the groups

Due to the high risk of submucosal invasion in cases with macroscopic progression, risk factors associated with macroscopic progression were investigated. There were no significant differences in macroscopic type, location, and size of lesions between patients with and without macroscopic progression (Table 3, Figure 4). In addition, none of the three patients with an initial histologic assessment of category 4 demonstrated apparent signs of macroscopic tumor progression during a median follow‐up of 27.0 (23.7–28.7) months. Rates of macroscopic progression at 150 months after detection were 11.1%, 16.7%, and 30.0% for SNADETs < 5mm, <10 mm, and ≥10 mm, respectively (Figure 5).

**TABLE 3:** Risk factors associated with macroscopic progression

	No growth *n* = 78	Macroscopic progression *n* = 8	OR (95% CI)	*p* value
**Patient factors**				
Age	65.8 ± 12.1	61.8 ± 17.2	NA	0.449
Gender				0.722
Female *n* (%)	35 (44.9%)	2 (25.0%)	1.00 (Control)	
Male *n* (%)	43 (55.1%)	6 (75.0%)	1.52 (0.30–9.76)	
**Lesion factors**				
Macroscopic type				0.775
0‐I	12 (15.4%)	3 (37.5%)	1.92 (0.32–9.42)	
0‐IIa	59 (75.6%)	4 (50.0%)	1.00 (Control)	
0‐IIc	7 (9.0%)	1 (12.5%)	0.64 (0.00–8.91)	
Location				0.720
Bulbus	13 (16.7%)	3 (37.5%)	1.42 (0.13–9.23)	
SDA*	4 (5.1%)	1 (12.5%)	4.10 (0.33–36.79)	
2nd portion	61 (78.2%)	4 (50.0%)	1.00 (Control)	
Lesion size				0.279
<10 mm	47 (60.3%)	2 (25.0%)	1.00 (Control)	
≧10 mm	31 (39.7%)	6 (75.0%)	2.48 (0.50–15.97)	
Biopsy result**				1.000
Category 3	75 (96.2%)	6 (100.0%)	1.00 (Control)	
Category 4	3 (3.8%)	0 (0.0%)	2.10 (0.01–32.81)	

All *p*‐values and confidence intervals were based on the penalized profile likelihood‐ratio test.

* SDA, superior duodenal angle.

** 2 excluded due to no biopsy or category 1. Although the odds ratio was estimated through Firth's penalized likelihood, the values should be interpreted with caution due to minimal or no event in this category.

## DISCUSSION

To the best of the authors’ knowledge, this is the largest cohort study with the longest follow‐up period describing the natural history of SNADETs to date. In this study, 4.6% of sporadic duodenal adenomas had confirmed progression to adenocarcinoma during a median follow‐up period of 36.8 (6.0–613.0) months. This suggests that the risk of duodenal adenomas developing to invasive cancer is low, which concurs with the only previous report on the natural course of SNADETs.^8^

However in clinical medicine, once treatment is postponed, it is often postponed indefinitely until there are clear indications for treatment. This is the first report demonstrating that in cases where immediate resection of SNADETs is not performed, changes in macroscopic size or shape strongly suggest carcinogenesis, with a high risk of submucosal invasion. These results concur with previous reports that changes in macroscopic size and shape are associated with submucosal invasive cancer in other gastrointestinal lesions.^19^, ^20^

Biopsy‐based endoscopic follow‐up has previously been suggested as an alternative strategy to endoscopic evaluation for the early detection of tumor progression.^8^ However, the efficacy of this strategy could not be confirmed in this study. Recent studies have demonstrated that the accuracy of histologic diagnoses of duodenal tumors based on endoscopic biopsies is limited.^12^, ^13^ The results of this study concur with these reports and suggest that clinical decisions based on upgrades in biopsy results may not be advocated.

Thus, although a wait‐and‐see strategy may be unavoidable for some cases of SNADETs, once this strategy is adopted, early detection of tumor progression is difficult. Especially in patients with no contraindications for resection, this strategy is accompanied with a substantial risk of progression to invasive cancer, regardless of macroscopic type and location or size of the lesions. Eventual surgical treatment, resulting in higher invasiveness than if the lesion was immediately treated with endoscopic resection, may be required. There are currently no guidelines concerning either the surveillance or treatment of SNADETs, and this study may provide evidence as a basis for future guidelines.

There were several limitations to our study. First, this was a single‐center retrospective study with a limited number of cases. However, the prevalence of SNADETs is low, and this is the largest cohort study describing the natural course of SNADETs to date. Second, selection bias cannot be ruled out. Although a recommendation of endoscopic resection was confirmed in the clinical records of all patients without contraindications, there is a possibility that endoscopic resection may not have been strongly recommended in all cases. Third, due to the nature of this study, long‐term endoscopic follow‐up was not always feasible due to major comorbidities. Both the incidence of tumor progression and period to tumor progression in this study should be referred to with caution. Fourth, this study was based on upper GI endoscopic diagnoses, which may have been affected by endoscopist expertise, and patient factors. In addition, as deep duodenal insertion is not always possible in upper GI endoscopic examinations, there is also a possibility that duodenal lesions, especially located in the third portion may have been overlooked. However, all endoscopic procedures were performed by senior endoscopists with over 3 years of training in upper GI endoscopy. Fifth, regular biopsy follow‐up was not performed in all cases. This may be one of the reasons the efficacy of biopsy‐based endoscopic follow‐up could not be confirmed in this study, and further research in this area is required.

## CONCLUSION

In the natural history of SNADETs, the overall risk of progression to invasive cancer is low. However, changes in macroscopic size or shape of SNADETs signify a high risk of progression to invasive cancer.

## CONFLICT OF INTEREST

All authors have no competing interests to declare.

## FUNDING INFORMATION

None.
